# Reliability and Diagnostic Performance of Digital Versus Clinical Assessment of Oral Hygiene and Gingival Health in Older Community‐dwelling Adults

**DOI:** 10.1111/scd.70268

**Published:** 2026-10-08

**Authors:** Isabel Deeg, Christoph Matthias Schoppmeier, Karolin Höfer, Alicia Maria Meyer‐Hofmann, Dirk Bleiel, Anna Greta Barbe

**Affiliations:** ^1^ Faculty of Medicine and University Hospital Cologne, Polyclinic For Operative Dentistry and Periodontology University of Cologne Cologne Germany; ^2^ Joint dental practice Dres. Pia und Dirk Bleiel Rheinbreitbach Germany

**Keywords:** aged, dental plaque, gingivitis, intraoral scanners, oral hygiene, telemedicine

## Abstract

**Aims:**

This study evaluated the diagnostic performance and interexaminer reliability of digital versus clinical oral hygiene assessments in community‐dwelling older adults, focusing on the Plaque Index for Long‐Term Care (PI‐LTC), Quigley–Hein Index (QHI), and Marginal Gingival Index (MGI).

**Methods and Results:**

Fifty‐two participants aged ≥ 65 years (mean 77.2 ± 6.8 years) underwent standardized clinical and digital examinations before (PI‐LTC, QHI, MGI) and after professional toothbrushing (QHI). Two independent digital examiners—one calibrated for gerodontology (D_1_) and one uncalibrated (D_2_)—assessed intraoral scans obtained with a 3Shape TRIOS 4 system (Straumann). Inter‐ and intraexaminer reliability were determined using intraclass correlation coefficients (ICC), and discriminative performance was analyzed via area under the receiver‐operating characteristic curve (AUROC). Both PI‐LTC and QHI demonstrated strong discrimination (AUROC 0.85–1.00). The QHI showed the highest and most consistent reliability (ICC ≈ 0.65–0.75), whereas the PI‐LTC achieved higher sensitivity but moderate reproducibility (ICC ≈ 0.50), particularly in posterior regions. The MGI yielded fair to good reliability (ICC ≈ 0.45–0.55) and was more examiner dependent. The calibrated examiner (D_1_) achieved near‐perfect agreement with the clinical reference (D_0_), while the uncalibrated examiner (D_2_) also performed acceptably but with lower accuracy (ΔAUROC ≈ 0.10–0.20).

**Conclusion:**

Digital intraoral scanning enables reliable and valid assessment of oral hygiene in older adults. Examiner calibration substantially enhances diagnostic precision, though uncalibrated digital evaluations also remain clinically useful. The QHI offers superior reproducibility, whereas the PI‐LTC provides greater sensitivity for plaque risk detection.

**Clinical Trial Registration:**

German Clinical Trials Register (DRKS), DRKS00032639, 19.07.2024

## 1 Introduction

Over recent decades, continuous improvements in oral health care and prevention have enabled an increasing proportion of older adults to retain their natural dentition throughout life [1, 2, 3, 4]. However, maintaining adequate daily oral hygiene can become progressively challenging With advancing age, particularly when functional impairments or cognitive decline occur [5]. This challenge is accentuated when individuals remain in their home environment Despite developing care dependency. In such community‐dwelling older adults, oral hygiene routines often rely on self‐management or informal caregiving by family members, who are typically untrained in professional oral care practices [6]. Under these circumstances, oral hygiene frequently becomes secondary to other aspects of daily care [7], and access to regular dental services is markedly limited. Moreover, organized outreach dental care remains scarce in most regions [2]

Consequently, gingivitis and moderate‐to‐severe periodontitis—Characterized by inflammation, pocket formation, and progressive attachment loss—Are highly prevalent in this population [8]. Beyond the evident impact on oral function, discomfort, and quality of life, compromised periodontal health in frail or care‐dependent older adults has substantial systemic implications. Accumulating evidence links poor oral hygiene and periodontal inflammation to increased risks of malnutrition, aspiration pneumonia, cardiovascular and neurodegenerative diseases, and a generally elevated systemic inflammatory burden [9, 10, 11, 12, 13, 14]. These interrelationships underline the urgent need for effective prevention, regular assessment, and early intervention strategies in geriatric oral health care, also outside of institutions of nursing homes. Despite this high demand, conventional dental prevention programs and recall‐based care models fail to adequately reach older adults in need of assistance. The traditional practice‐based approach is often impractical due to mobility limitations, transportation barriers, and a lack of structured collaboration Between dental professionals, general healthcare providers, and nursing personnel [15, 16, 17]. A critical gap therefore persists in the development of validated, feasible tools that support interprofessional communication, enable objective assessment of oral hygiene conditions in community settings, and document the effects of prophylactic or supportive interventions Over time

In future domiciliary care concepts, digital intraoral documentation could potentially be performed not only by dental professionals During outreach visits, but also by non‐dental personnel involved in everyday geriatric care, such as home‐care nurses, nursing assistants, or trained family caregivers. The acquired scan data could subsequently be reviewed remotely by dental professionals for screening, monitoring, and quality assurance purposes, thereby facilitating interdisciplinary collaboration Between dentistry, nursing care, and informal caregiving structures in community‐based settings. However, for such approaches to become clinically meaningful and practically applicable, reliable and reproducible methods for the assessment of oral hygiene and gingival health are required. In particular, it remains unclear whether visual or digitally supported methods, such as intraoral scanning, provide sufficient validity and reproducibility for these settings and which oral hygiene indices are suitable for non‐clinical domiciliary environments

To address these challenges, the present study focused on three indices With specific methodological advantages for geriatric domiciliary assessment. The Plaque Index for Long‐Term Care (PI‐LTC) was selected because it is a validated index specifically established for geriatric and long‐term care settings and can be assessed purely visually Without any invasive intervention [18]. The Modified Gingival Index (MGI) was chosen as a likewise exclusively visual parameter for gingival inflammation that has already demonstrated good applicability in previous scan‐based studies [19, 20]. In contrast, the Quigley–Hein Index (QHI) was primarily included to enable enhanced biofilm quantification During professionally assisted toothbrushing procedures, as plaque disclosure substantially improves visualization and assessment on digital intraoral scans [21]. Together, these indices were intended to cover complementary dimensions of oral hygiene and gingival health while remaining feasible for digital scan and outreach‐based application

Although intraoral scanners are not yet routinely available for domiciliary dental visits and are not primarily developed for outreach dental care, technological progress is rapidly reducing the logistical and financial barriers to their use

Previous studies have demonstrated that intraoral scanners can be used not only for restorative procedures but also for oral health assessment. Doi et al. [22]. reported that digital scans can facilitate plaque detection and oral hygiene instruction and may reveal plaque deposits that are difficult to identify During direct clinical examination. Systematic evaluations of scanning technology have further demonstrated high levels of accuracy and reproducibility for intraoral surface acquisition [23, 24], supporting their potential use for diagnostic applications Beyond prosthodontics. More recently, Kharbot et al. [25]. showed that intraoral scans allow reliable assessment of oral hygiene parameters in nursing home residents. However, their investigation focused exclusively on the Geriatric Debris Index Simplified (GDI‐S), which evaluates selected buccal tooth surfaces only, and did not include assessment of gingival health. In contrast, the present study evaluated both oral hygiene and gingival health using three established indices (PI‐LTC, QHI, and MGI), included assessment of all clinically visible tooth surfaces. Furthermore, the study focused on community‐dwelling older adults, a population that has received comparatively little attention in previous digital oral health assessment research

Moreover, it remains unclear whether calibrated and uncalibrated examiners achieve comparable agreement in assessing oral hygiene indices on digital scans—An essential prerequisite if such assessments are to be delegated or validated remotely, for example, Within general dental practices for quality assurance of nursing‐based prophylaxis activities. To address these knowledge gaps, the present monocentric, prospective cohort study investigated community‐dwelling older adults aged ≥65 years. For each participant, established oral hygiene and gingival health indices were recorded using visual assessment and plaque‐disclosing methods, and digital intraoral scans were obtained Before and After professional toothbrushing. These assessments and scans took place in a dental clinic. Two independent examiners—One calibrated for gerodontology and one non‐calibrated for gerodontology—Performed digital index scoring on standardized scan visualizations to evaluate intra‐ and interexaminer agreement, as well as potential effects on biofilm amount of professional brushing. The study thus aims to explore the feasibility, reliability, and diagnostic consistency of digital oral hygiene assessment in older adults living at home, providing a methodological foundation for future interdisciplinary preventive strategies in geriatric oral health care

## 2 Materials and Methods

The study received ethical approval from the local ethics review board and was registered prior to patient enrollment (23‐1060). All procedures complied with the ethical standards of the institutional research committee, the 1964 Helsinki Declaration, and subsequent amendments or comparable ethical standards. Informed consent was obtained from all participants or their legal guardians. The data that support the findings of this study are available from the corresponding author upon reasonable request.

### 2.1 Study Population

Participants were recruited from the patient cohort of the Department of Operative Dentistry and Periodontology at University of Cologne, Cologne, Germany from 08/2023 to 02/2024. Eligible individuals were community‐dwelling adults aged 65 years or older who presented with at least four remaining natural teeth and exhibited a Plaque Index for Long‐Term Care (PI‐LTC) greater than zero, indicating the presence of plaque accumulation requiring professional evaluation. Individuals were excluded if they did not provide written informed consent, exhibited an insufficient ability to open the mouth for clinical examination or scanning, or had fewer than four residual teeth or a foreseeable loss of the remaining dentition during the study period.

### 2.2 Sample Size

The sample size was determined a priori based on the primary outcome variable, the PI‐LTC, with the objective of estimating the interclass correlation coefficient (ICC) for repeated assessments. Assuming an expected ICC of 0.75 and a desired 95% confidence interval width of 0.20, a minimum of 52 participants was calculated to be necessary when three independent examiners (one on‐site clinical assessment and two digital evaluations based on intraoral scans) were included. A total of 54 individuals were screened for participation. Of these, 52 participants were included in the study, whereas two individuals could not be enrolled due to time constraints and a temporary inpatient rehabilitation stay, respectively. All examinations and digital assessments were conducted by the same three dentists to ensure consistency across evaluations.

### 2.3 Study Protocol and Procedures

All participants were informed about the study both verbally and in writing during their routine dental visit at the Department of Operative Dentistry and Periodontology, University of Cologne, Cologne, Germany. Written informed consent was obtained from all participants prior to inclusion. The study procedures were fully integrated into routine dental care, and all assessments were performed chairside under standard clinical conditions by qualified dental personnel. Following completion of the standardized clinical examination, a series of intraoral assessments were conducted to capture oral hygiene indices and digital images at different time points. The Plaque Index for Long‐Term Care (PI‐LTC) and Modified Gingival Index (MGI) and clinical examinations were recorded before plaque disclosing. Plaque disclosing was performed using Mira 2‐Ton tablets (Miradent, Hager Pharma GmbH, Duisburg, Germany), a routinely used and non‐invasive method that enables visualization of fresh and mature plaque layers. After plaque disclosing Quigley‐Hein Index (QHI) was obtained, followed by professional brushing. After professional brushing the QHI was recorded a second time. Intraoral scans of both jaws were obtained at each assessment point using a 3Shape TRIOS 4 scanner (e.g. TRIOS 4, 3Shape, Copenhagen, Denmark), operated by a dental assistant trained to at least the qualification level of a registered dental nurse (Zahnmedizinische Fachangestellte in Germany). Scans were conducted in accordance with the manufacturer's Safety and Setup Guide (CE‐certified device). Professional toothbrushing was then performed by a dental hygienist or dental nurse trained at this level, using an oscillating powered toothbrush and fluoridated toothpaste, as well as interdental brushes. This procedure simulated a realistic domiciliary oral care intervention. All procedures were non‐invasive and corresponded to standard dental prophylactic measures. The entire appointment, including the clinical assessment, scanning, disclosing, and professional toothbrushing, lasted approximately 30 min per participant.

### 2.4 Study Outcomes and Assessment Procedures

The Primary Outcome Measure of this Investigation Was the Level of Agreement for the Plaque Index for Long‐Term Care (PI‐LTC) [18] Between Clinical Assessment and Digital Evaluation Based on Intraoral Scans. The PI‐LTC Was Recorded Within a Range From 0 to 3, With Higher Values Indicating Poorer Oral Hygiene. Agreement Between Clinical and Digital Assessments Was Evaluated to Determine the Reliability and Potential Interchangeability of both Methods Across Examiners

Secondary Outcome Measures Included the Modified Gingival Index (MGI; Range: 0–4) [19] Reflecting the Visually Detectable Degree of Gingival Inflammation, the Quigley‐Hein‐Index (QHI; Range 0 – 5) [21, 26] Displaying Disclosed Biofilm Accumulation Before and After Professional Brushing, and a Standardized Clinical Examination Documenting Also Additional Clinical Characteristics. Demographic and General Health‐related Parameters Were Also Collected, Including Age, Sex, Decayed, Missing, and Filled Teeth (DMFT) Index, Current Medication List, Care Dependency Level, and Comprehensive Dental and Medical History

### 2.5 Oral Scans

For digital evaluation, full‐arch intraoral scans were acquired using a high‐resolution optical scanner (e.g. TRIOS 4, 3Shape, Copenhagen, Denmark) based on confocal imaging technology and in accordance with the manufacturer's instructions. Scanning was performed in the clinical setting of a dental clinic under uniform ambient lighting conditions, avoiding direct illumination of the oral cavity, and after gentle air drying of the teeth. A standardized, predefined scan path was followed, beginning with the occlusal surfaces of the full arches from the patient's distal left side. In the anterior region, controlled pivoting movements were applied to compensate for the limited surface morphology of the incisal edges. In the maxilla, scanning proceeded from the buccal surfaces in the opposite direction before continuing onto the palatal surfaces and palate, again starting from the distal left side. In the mandible, the lingual surfaces were scanned first, followed by the buccal surfaces. All scans were performed by the same calibrated dental prophylaxis assistant with participants seated in an upright position and were completed within approximately 5–7 min per participant, ensuring comprehensive capture of all tooth surfaces and gingival margins. The resulting digital datasets were anonymized and independently assessed by two examiners—one calibrated and one non‐calibrated—using standardized visualization software.

### 2.6 Professional Brushing

Professional Toothbrushing Was Performed Immediately Following the Baseline Assessments by One Trained Dental Hygienist Using a Powered Oscillating Toothbrush (Oral‐B Genius X, Oral‐B Pro CrossAction Replacement Brush Head, Procter&Gamble, Cincinnati, Ohio, Usa) and Fluoridated Toothpaste (Dentagard, Colgate, New York City, Usa). The Brushing Procedure Was Standardized to Simulate a Realistic Domiciliary Care Intervention: All Accessible Tooth Surfaces Were Cleaned for a Total Duration of Approximately 2 Minutes. Interproximal Cleaning Was Caried out Using Interdental Brushes in Patient Individually Adjusted Sizes (Tepe, Malmö, Sweden). No Adjunctive Professional Instruments (E.g., Ultrasonic Scalers) Were Used, Ensuring Comparability With Daily Care Routines in Community‐based or Nursing Home Settings. After Brushing, the Intraoral Scan and Clinical Assessments Were Repeated to Evaluate Changes in Plaque Indices and to Determine the Responsiveness of the QHI to Routine Prophylactic Measures

### 2.7 Image Evaluation

All digital evaluations were performed under standardized and controlled conditions. The anonymized intraoral scan images were assessed in a quiet, dimly lit room free from external distractions to ensure optimal viewing conditions. Examiners used the scanner screen attached with neutral background settings in order to simulate a telemedical setting. Each examiner independently reviewed the digital scans in full‐screen mode without time constraints, ensuring systematic and reproducible scoring of the plaque and gingival indices.

### 2.8 Examiner Calibration and Expertise

Two independent examiners (D_1_ and D_2_) evaluated all intraoral scans. One examiner was closely calibrated with the clinical investigator prior to the study, reflecting a long‐standing collaboration through joint teaching and clinical activities as well as performing the same specialization in gerodontology. Calibration was confirmed by achieving >90% agreement in the scoring of 10 representative images assessed independently before study initiation. This examiner held a certified specialization in geriatric dentistry, having completed an accredited postgraduate curriculum in senior oral healthcare, and possessed extensive clinical experience in assessing oral hygiene status in older adults.

The second examiner, by contrast, received no specific calibration or prior training especially for this study. However, the examiner was provided with a standardized scoring protocol including both textual descriptions and visual example illustrations of the applied indices prior to assessment of the digital scans. This examiner was not specialized in gerodontology but was a board‐certified pediatric dentist with recognized expertise in preventive and prophylactic care in children. The inclusion of these two examiners with very distinct clinical backgrounds and calibration statuses was intended to evaluate the robustness, reproducibility, and potential generalizability of digital plaque assessments across varying levels of specialization and training.

### 2.9 Statistical Analysis

All analyses were conducted using IBM SPSS Statistics (Version 30, IBM Corp., Armonk, NY, USA). Descriptive statistics summarized all clinical and demographic variables. Continuous data are presented as mean ± standard deviation (SD) and categorical data as absolute and relative frequencies. Normality of distributions was assessed using the Shapiro–Wilk test. Inter‐ and intraexaminer reliability for the Plaque Index for Long‐Term Care (PI‐LTC), Modified Gingival Index (MGI), and Quigley–Hein Index (QHI) were quantified using intraclass correlation coefficients (ICC) with 95% confidence intervals (CI). ICC(2) (two‐way random effects, absolute agreement) and ICC(3) (two‐way mixed effects, consistency) models were applied according to whether raters were considered random or fixed effects. Reliability was interpreted as poor (<0.40), fair (0.40–0.59), good (0.60–0.74), or excellent (≥0.75) in accordance with Cicchetti criteria [27]. Differences in examiner agreement were assessed by comparing dependent ICC values [28]. To assess discriminative performance, receiver operating characteristic (ROC) analyses were performed, and areas under the curve (AUROC) were calculated for each index and anatomical region. AUROC values were interpreted as follows: ≥0.90 excellent, 0.80–0.89 good, and 0.70–0.79 acceptable discrimination. Sensitivity, specificity, and CI values were derived for each examiner and presented in tabular form. Pre–post comparisons of QHI values were evaluated with paired t‐tests or Wilcoxon signed‐rank tests, as appropriate. The mean change (ΔQHI) was calculated to estimate plaque reduction after professional toothbrushing. All analyses were two‐tailed, and p < 0.05 was considered statistically significant.

## 3 Results

### 3.1 Clinical Characteristics

A total of 52 community‐dwelling older adults (mean age 77.2 ± 6.8 years; range, 65–94 years) were included. Participants presented with a mean of 2.9 ± 2.6 systemic conditions and reported 4.4 ± 4.2 regularly taken medications. The dentition was generally well maintained, with an average of 10.6 ± 6.2 missing, 13.3 ± 5.4 filled teeth and 0.13 ± 0.44 decayed teeth (Table 1).

**TABLE 1:** Clinical and oral characteristics of the study participants (*n* = 52).

Variable	*N*	Mean	SD	Median	Minimum	Maximum
Age (years)	52	77.15	6.83	77.5	65.0	94.0
Number of diseases	52	2.9	2.61	3.0	0.0	16.0
Number of medications	52	4.44	4.18	4.0	0.0	19.0
Missing teeth	52	10.63	6.17	9.0	0.0	26.0
Filled teeth	52	13.27	5.42	13.0	2.0	29.0
Decayed teeth	52	0.13	0.44	0.0	0.0	2.0
PILTC^a^ D0 total	52	0.56	0.44	0.46	0.0	2.3
PILTC^a^ D1 total	52	0.58	0.4	0.44	0.06	1.9
PILTC^a^ D2 total	52	0.75	0.45	0.7	0.0	2.58
QHI^b^ pre D0 total	52	1.63	0.69	1.54	0.12	3.12
QHI^b^ pre D1 total	51	1.7	0.68	1.74	0.52	3.33
QHI^b^ pre D2 total	52	1.53	0.53	1.5	0.26	3.16
QHI^b^ post D0 total	52	0.01	0.02	0.0	0.0	0.11
QHI^b^ post D1 total	52	0.03	0.04	0.0	0.0	0.15
QHI^b^ post D2 total	52	0.16	0.22	0.1	0.0	1.13
ΔQHI^c^ D0 total	52	−1.6	0.65	−1.57	−3.0	−0.2
ΔQHI^c^ D1 total	52	−1.62	0.68	−1.62	−2.91	−0.3
ΔQHI^c^ D2 total	52	−1.34	0.52	−1.27	−2.33	−0.05
MGI^d^ D0 total	52	1.03	0.72	0.91	0.06	3.15
MGI^d^ D1 total	52	1.03	0.63	0.88	0.17	3.25
MGI^d^ D2 total	51	0.58	0.5	0.46	0.03	2.69
PBI^e^ D0 total	52	1.32	0.73	1.23	0.19	3.6

Notes: Clinical, medical, and oral hygiene characteristics of the study participants. Dental and periodontal parameters were assessed independently by three examiners (D0, D1, D2). D0 = clinical examiner (reference); D1 = calibrated digital examiner; D2 = non‐calibrated digital examiner. The following indices were recorded: Plaque Index for Long‐Term Care (PILTC), Quigley‐Hein Index (QHI) before (pre) and after (post) professional brushing procedures, change in QHI (ΔQHI), Modified Gingival Index (MGI), and Papilla Bleeding Index (PBI). Data are presented as mean, standard deviation (SD), median, minimum, and maximum values.

^a^ PILTC = Plaque Index for Long Term Care.

^b^ QHI = Quigley‐Hein Index.

^c^ ΔQHI = Change in QHI from pre‐ to post‐professional brushing.

^d^ MGI = Modified Gingival Index.

^e^ PBI = Papilla Bleeding Index.

### 3.2 Baseline Oral Hygiene and Clinical Indices

At baseline, total documented Plaque Index for Long‐Term Care (PI‐LTC) values ranged from 0.56 ± 0.44 (D_0_) to 0.75 ± 0.45 (D_2_), with medians between 0.46 and 0.70. The Modified Gingival Index (MGI) averaged approximately 1.0 for D_0_ and D_1_ and 0.58 ± 0.50 for D_2_, indicating mild to moderate gingival inflammation. The Papillary Bleeding Index (PBI) measured by the clinical examiner (D_0_) was 1.32 ± 0.73, confirming localized bleeding and low‐grade gingival inflammation typical for this population.

### 3.3 Examiner‐specific Findings and QHI Response to Brushing

Across clinical and digital examiners, mean PI‐LTC and MGI scores were consistent, suggesting good interexaminer alignment. Baseline Quigley–Hein Index (QHI) values indicated moderate plaque presence (D_0_ = 1.63 ± 0.69; D_1_ = 1.70 ± 0.68; D_2_ = 1.53 ± 0.53). Following professional toothbrushing, QHI values declined markedly to near‐zero for D_0_ (0.01 ± 0.02) and D_1_ (0.03 ± 0.04), and to 0.16 ± 0.22 for D_2_. The mean change (ΔQHI) confirmed a strong intervention effect (D_0_ = −1.60 ± 0.65; D_1_ = −1.62 ± 0.68; D_2_ = −1.34 ± 0.52), reflecting substantial plaque removal across all assessments.

### 3.4 Interexaminer Agreement and Reliability

Interexaminer reliability for PI‐LTC was generally moderate to excellent depending on the region. The strongest agreement between digital and clinical assessments occurred in molar and premolar regions (D_2_: ICC = 0.83 and 0.77; D_1_: ICC = 0.81 and 0.59), consistent with excellent or good reliability. Anterior and vestibular surfaces yielded moderate‐to‐good ICCs (0.57–0.73), while isolated oral molar sites showed weaker correspondence (ICC < 0.40), likely due to methodological challenges affecting both clinical inspection and digital image acquisition. In particular, posterior molar regions were generally more difficult to access and visualize during both examination and scanning procedures. In many older participants, increased cheek muscle tone, gag reflex and limited oral opening further complicated scanner positioning and intraoral visibility in the posterior oral regions. In addition, illumination in these distal areas was inherently more restricted, which may have negatively influenced both direct visual assessment and digital scan quality. For MGI, examiner agreement between D_0_ and D_1_ was consistently good (ICC ≈ 0.51–0.56), whereas D_2_ exhibited lower reliability (ICC ≈ 0.43–0.50) and systematically rated less gingival inflammation, consistent with the examiner's non‐calibrated status. For QHI, both ICC(2) and ICC(3) values exceeded 0.70 across most regions, denoting excellent reproducibility. After brushing, ΔQHI retained high reliability (ICC(2) = 0.64), confirming consistent detection of plaque reduction among examiners (Table 2, Supporting material).

**TABLE 2:** Inter‐examiner reliability and descriptive statistics of PILTC, QHI, and MGI scores.

Variable	Median	SD	ICC2	ICC3
**PILTC**				
PILTC^a^ D0 upper jaw	0.22	0.31	0.436	0.502
PILTC^a^ D1 upper jaw	0.28	0.30		
PILTC^a^ D2 upper jaw	0.59	0.34		
PILTC^a^ D0 lower Jaw	0.57	0.20	0.374	0.465
PILTC^a^ D1 lower Jaw	0.79	0.20		
PILTC^a^ D2 lower Jaw	0.79	0.20		
PILTC^a^ D0 oral	0.54	0.18	0.402	0.533
PILTC^a^ D1 oral	0.81	0.19		
PILTC^a^ D2 oral	0.81	0.19		
PILTC^a^ D0 oral front	0.58	0.42	0.516	0.544
PILTC^a^ D1 oral front	0.50	0.40		
PILTC^a^ D2 oral front	0.82	0.43		
PILTC^a^ D0 oral molars	0.70	7.06	−0.022	−0.022
PILTC^a^ D1 oral molars	0.80	0.63		
PILTC^a^ D2 oral molars	1.00	0.43		
PILTC^a^ D0 oral premolars	0.60	0.48	0.500	0.524
PILTC^a^ D1 oral premolars	0.60	0.46		
PILTC^a^ D2 oral premolars	0.85	0.43		
PILTC^a^ D0 buccal	0.29	0.35	0.533	0.592
PILTC^a^ D1 buccal	0.59	0.34		
PILTC^a^ D2 buccal	0.59	0.34		
PILTC^a^ D0 buccal front	0.28	0.41	0.571	0.612
PILTC^a^ D1 buccal front	0.33	0.42		
PILTC^a^ D2 buccal front	0.67	0.37		
PILTC^a^ D0 buccal molarss	0.50	0.48	0.225	0.226
PILTC^a^ D1 buccal molars	0.60	0.49		
PILTC^a^ D2 buccal molars	0.75	0.46		
PILTC^a^ D0 buccal premolars	0.14	0.35	0.443	0.502
PILTC^a^ D1 buccal premolars	0.25	0.44		
PILTC^a^ D2 buccal premolars	0.50	0.42		
PILTC^a^ D0 total	0.46	0.44	0.533	0.560
PILTC^a^ D1 total	0.44	0.40		
PILTC^b^ D2 total	0.70	0.45		
**QHI**				
QHI^b^ pre D0 upper jaw	1.38	0.66	0.721	0.722
QHI^b^ pre D1 upper jaw	1.50	0.68		
QHI^b^ pre D2 upper jaw	1.29	0.61		
QHI^b^ pre D0 lower jaw	1.71	0.80	0.651	0.654
QHI^b^ pre D1 lower jaw	1.74	0.79		
QHI^b^ pre D2 lower jaw	1.61	0.65		
QHI^b^ pre D0 oral	1.62	0.76	0.639	0.641
QHI^b^ pre D1 oral	1.64	0.78		
QHI^b^ pre D2 oral	1.50	0.66		
QHI^b^ pre D0 oral front	1.77	0.80	0.664	0.666
QHI^b^ pre D1 oral front	1.58	0.80		
QHI^b^ pre D2 oral front	1.58	0.73		
QHI^b^ pre D0 oral molars	1.62	0.76	0.625	0.625
QHI^b^ pre D1 oral molars	1.70	0.99		
QHI^b^ pre D2 oral molars	1.82	0.86		
QHI^b^ pre D0 oral premolars	1.40	0.88	0.546	0.547
QHI^b^ pre D1 oral premolars	1.43	0.83		
QHI^b^ pre D2 oral premolars	1.38	0.73		
QHI^b^ pre D0 buccal	1.51	0.72	0.720	0.727
QHI^b^ pre D1 buccal	1.60	0.73		
QHI^b^ pre D2 buccal	1.43	0.65		
QHI^b^ pre D0 buccal front	1.52	0.80	0.723	0.726
QHI^b^ pre D1 buccal front	1.44	0.78		
QHI^b^ pre D2 buccal front	1.33	0.65		
QHI^b^ pre D0 buccal molars	1.57	0.87	0.667	0.668
QHI^b^ pre D1 buccal molars	1.83	0.97		
QHI^b^ pre D2 buccal molars	1.58	0.86		
QHI^b^ pre D0 buccal premolars	1.43	0.73	0.613	0.636
QHI^b^ pre D1 buccal premolars	1.62	0.83		
QHI^b^ pre D2 buccal premolars	1.21	0.72		
QHI^b^ pre D0 total	1.54	0.69	0.741	0.750
QHI^b^ pre D1 total	1.74	0.68		
QHI^b^ pre D2 total	1.50	0.53		
QHI^b^ post D0 oral front	0.00	0.03	0.008	0.009
QHI^b^ post D1 oral front	0.00	0.05		
QHI^b^ post D2 oral front	0.00	0.26		
QHI^b^ post D0 oral molars	0.00	0.05	0.095	0.115
QHI^b^ post D1 oral molars	0.00	0.17		
QHI^b^ post D2 oral molars	0.11	0.68		
QHI^b^ post D0 oral premolars	0.00	0.06	0.050	0.059
QHI^b^ post D1 oral premolars	0.00	0.05		
QHI^b^ post D2 oral premolars	0.00	0.38		
QHI^b^ post D0 buccal front	0.00	0.14	−0.041	−0.045
QHI^b^ post D1 buccal front	0.00	0.05		
QHI^b^ post D2 buccal front	0.00	0.23		
QHI^b^ post D0 buccal molars	0.00	0.03	0.031	0.038
QHI^b^ post D1 buccal molars	0.00	0.26		
QHI^b^ post D2 buccal molars	0.17	0.67		
QHI^b^ post D0 buccal premolars	0.00	0.03	0.115	0.129
QHI^b^ post D1 buccal premolars	0.00	0.08		
QHI^b^ post D2 buccal premolars	0.00	0.28		
QHI^b^ post D0 total	0.00	0.02	0.033	0.047
QHI^b^ post D1 total	0.00	0.04		
QHI^b^ post D2 total	0.10	0.22		
ΔQHI^c^ D0 total	−1.57	0.65	0.640	0.677
ΔQHI^c^ D1 total	−1.62	0.68		
ΔQHI^c^ D2 total	−1.27	0.52		
**MGI**				
MGI^d^ D0 upper jaw	0.76	0.72	0.547	0.657
MGI^d^ D1 upper jaw	0.82	0.72		
MGI^d^ D2 upper jaw	0.38	0.43		
MGI^d^ D0 lower jaw	0.90	0.69	0.511	0.639
MGI^d^ D1 lower jaw	0.86	0.66		
MGI^d^ D2 lower jaw	0.50	0.30		
MGI^d^ D0 oral	0.87	0.71	0.508	0.650
MGI^d^ D1 oral	0.90	0.65		
MGI^d^ D2 oral	0.34	0.41		
MGI^d^ D0 oral front	0.73	0.68	0.563	0.657
MGI^d^ D1 oral front	0.76	0.65		
MGI^d^ D2 oral front	0.33	0.44		
MGI^d^ D0 oral molars	1.00	0.75	0.438	0.627
MGI^d^ D1 oral molars	1.12	0.72		
MGI^d^ D2 oral molars	0.40	0.48		
MGI^d^ D0 oral premolars	1.00	0.71	0.438	0.577
MGI^d^ D1 oral premolars	0.86	0.65		
MGI^d^ D2 oral premolars	0.35	0.42		
MGI^d^ D0 buccal	0.91	0.71	0.519	0.600
MGI^d^ D1 buccal	0.82	0.71		
MGI^d^ D2 buccal	0.54	0.38		
MGI^d^ D0 buccal front	0.75	0.78	0.548	0.602
MGI^d^ D1 buccal front	0.67	0.77		
MGI^d^ D2 buccal front	0.59	0.44		
MGI^d^ D0 buccal molars	1.00	0.65	0.462	0.538
MGI^d^ D1 buccal molars	1.00	0.65		
MGI^d^ D2 buccal molars	0.50	0.56		
MGI^d^ D0 buccal premolars	0.88	0.74	0.478	0.563
MGI^d^ D1 buccal premolars	0.80	0.70		
MGI^d^ D2 buccal premolars	0.50	0.39		
MGI^d^ D0 total	0.91	0.72	0.508	0.594
MGI^d^ D1 total	0.88	0.63		
MGI^d^ D2 total	0.46	0.50		

Notes: Descriptive statistics and inter‐examiner reliability of oral health indices assessed independently by three examiners (D0, D1, D2); D0 = clinical examiner (reference); D1 = calibrated digital examiner; D2 = non‐calibrated digital examiner. The Plaque Index for Long‐Term Care (PILTC), Quigley‐Hein Plaque Index (QHI) before and after professional brushing procedures, change in QHI (ΔQHI), and the Modified Gingival Index (MGI) were recorded for different jaw regions and tooth groups. Data are presented as mean, standard deviation (SD), median, minimum, maximum, and intraclass correlation coefficients (ICC2 and ICC3).

ICC2 = two‐way random‐effects model, absolute agreement.

ICC3 = two‐way mixed‐effects model, consistency.

SD = standard deviation.

According to Cicchetti1, intraclass correlation coefficients were interpreted as follows and are visually coded in the table: ICC < 0.40 = poor reliability (red),

ICC 0.40–0.59 = fair reliability (yellow),

ICC 0.60–0.74 = good reliability (green),

ICC ≥ 0.75 = excellent reliability (dark green).

^a^ PILTC = Plaque Index for Long Term Care.

^b^ QHI = Quigley‐Hein Index.

^c^ ΔQHI = Change in QHI from pre‐ to post‐professional brushing.

^d^ MGI = Modified Gingival Index.

1. Cicchetti DV. Guidelines, criteria, and rules of thumb for evaluating normed and standardized assessment instruments in psychology. *Psychological Assessment* 1994;6:284–290.

### 3.5 Diagnostic Discrimination and Model Performance

Risk modeling revealed a strong discriminative capacity for both indices, though with distinct performance profiles. The plaque index percentage (PI‐%) cutoff values proposed by Azevedo et al. (2022) [29] were adapted to the Quigley–Hein Index (QHI) (cutoff value 2) and the Plaque Index for Long‐Term Care (PILTC) (cutoff value 1) by approximating plaque‐covered tooth surface areas, thereby enabling classification into health and risk for gingivitis categories. For the Plaque Index for Long‐Term Care (PI‐LTC), total and vestibular scores achieved AUROC values between 0.95 and 1.00, with corresponding sensitivities ≥ 0.85 and specificities ≥ 0.80, indicating near‐perfect discrimination between individuals with high and low plaque levels. The highest regional stability was observed for anterior and premolar segments (ICC ≈ 0.44–0.57), whereas molar regions exhibited greater variability, reflecting reduced reproducibility in posterior areas. In contrast, the Quigley–Hein Index (QHI) demonstrated uniformly high discrimination across all regions, with AUROC values ranging from 0.85 to 0.98 and balanced sensitivity and specificity. Interexaminer reliability for the QHI was superior (ICC ≈ 0.65–0.74), and aggregated scores for total and vestibular sites reached AUROC values close to 1.00, confirming the robustness of this index. Direct comparison of examiner performance showed that the calibrated digital examiner (D_1_) closely mirrored the clinical reference (D_0_), whereas the non‐calibrated examiner (D_2_) consistently produced lower AUROC values (Δ = 0.10–0.20) and reduced reliability. No significant AUROC differences were found between D_0_ and D_1_ (p > 0.05), underscoring the validity of calibrated digital assessment as a reliable surrogate for clinical evaluation (Table 3).

**TABLE 3:** Diagnostic performance and interexaminer reliability of PILTC and QHI models.

Variable	Examiner	AUROC	Sensitivity	Specificity	ICC
**PILTC**					
Total	D0	1.0	1.0	1.0	0.53
	D1	0.976	1.0	0.89	0.53
	D2	0.784	0.86	0.71	0.53
Buccal	D0	0.984	1.0	0.89	0.53
	D1	0.786	0.86	0.61	0.53
	D2	0.786	0.86	0.61	0.53
Oral front	D0	0.927	1.0	0.78	0.52
	D1	0.841	1.0	0.67	0.52
	D2	0.767	0.71	0.73	0.52
Oral premolars	D0	0.932	1.0	0.84	0.5
	D1	0.911	1.0	0.75	0.5
	D2	0.789	0.86	0.63	0.5
**QHI**					
Total (pre‐brushing)	D0	1.0	1.0	1.0	0.74
	D1	0.993	1.0	0.94	0.74
	D2	0.857	0.84	0.76	0.74
Buccal (pre‐brushing)	D0	0.88	0.9	0.79	0.72
	D1	0.87	0.79	0.91	0.72
	D2	0.786	0.63	0.82	0.72
Oral (pre‐brushing)	D0	0.96	1.0	0.79	0.64
	D1	0.912	0.79	0.97	0.64
	D2	0.809	0.63	0.94	0.64

Diagnostic accuracy and interexaminer reliability of PILTC and QHI models assessed by three examiners D0, clinical examiner (reference); D1, calibrated digital examiner; D2, non‐calibrated digital examiner. Receiver operating characteristic (ROC) analysis was used to determine the area under the ROC curve (AUROC), sensitivity, and specificity. Interexaminer reliability was evaluated using intraclass correlation coefficients (ICC). Other abbreviations: PI‐LTC, Plaque Index for Long Term Care; QHI, Quigley‐Hein Index; SD, standard deviation.

## 4 Discussion

The primary objective of this study was not to compare oral hygiene indices per se, but to determine whether digital intraoral scan–based assessment can provide results comparable to conventional clinical examination in older adults. Across all evaluated parameters, digital assessment demonstrated good‐to‐excellent agreement With the reference examination and high diagnostic discrimination. Furthermore, the overall pattern of findings was largely consistent Across examiners and indices, supporting the use of digital intraoral scans as a reliable basis for oral hygiene assessment in this population. Although differences Between individual indices were observed, these findings should be interpreted as secondary methodological observations rather Than as evidence Against the overall validity of scan‐based assessment. Examiner calibration further improved agreement and measurement precision, highlighting the importance of standardized assessment procedures for digital evaluation

The overall agreement Between digital and clinical assessments was not uniform Across all oral regions. Consistent With previous studies, anterior regions showed the highest reproducibility, whereas posterior molar areas were associated With lower agreement [22, 30]. These differences are likely attributable to anatomical and technical factors that affect both clinical inspection and scan acquisition, including restricted visibility, limited oral opening, gag reflexes, and reduced illumination in posterior regions. Importantly, these limitations affected both clinical and digital assessment methods and therefore do not fundamentally challenge the validity of scan‐based oral hygiene evaluation. Instead, they highlight areas where future improvements in scanner technology and imaging protocols may further enhance diagnostic performance

Besides anatomical accessibility and visibility constraints, technical factors related to scan acquisition may also have contributed to the reduced agreement observed in posterior regions. Intraoral scanning of distal molars is inherently challenging because of limited working space, soft tissue interference, and the increased complexity of image stitching in these areas. As scan quality metrics and stitching artefacts were not systematically assessed in the present study, the relative contribution of technical acquisition factors cannot be determined. Future investigations should evaluate scan quality parameters alongside diagnostic reliability to better understand their potential influence on assessment outcomes

Examiner calibration emerged as a central determinant of reliability and diagnostic validity. The calibrated digital examiner (D_1_) achieved performance metrics nearly identical to the clinical reference (D_0_), whereas the non‐calibrated examiner (D_2_) demonstrated lower ICC and AUROC values, typically by Δ = 0.10–0.20. Although all examiners were experienced dentists, D_0_ and D_1_ had greater routine exposure to gerodontological patients and oral hygiene assessment in older adults. Differences in clinical focus and patient populations may therefore have influenced scoring behaviour and interpretation thresholds. For example, frequent exposure to substantial plaque accumulation may have affected the perception and classification of minor plaque deposits. However, examiner experience and case exposure were not formally quantified in this study. Consequently, this explanation should be regarded as a plausible interpretation rather Than a proven causal mechanism. Despite this, uncalibrated digital assessments yielded acceptable results, suggesting that standardized visualization and scoring protocols can partially compensate for limited examiner calibration

### 4.1 Index‐specific interpretation

#### 4.1.1 Quigley–Hein Index (QHI)

The QHI exhibited the highest and most consistent reliability Across all indices, particularly in pre‐brushing conditions, where ICC values indicated good to excellent interrater agreement. This strong concordance reflects the high visual clarity of plaque After disclosing and the relative ease of differentiating stained surfaces on digital scans as shown earlier [25, 31, 32, 33, 34]. Following professional toothbrushing, however, the ICC dropped markedly, even reaching negative values in some regions. This decline does not indicate methodological failure but reflects a known limitation of the ICC when variance is low—Small absolute differences Between raters become disproportionally magnified once plaque levels approach zero which is expected After professional brushing. Interestingly, the uncalibrated examiner (D2) consistently reported slightly higher residual QHI scores following professional toothbrushing compared With the calibrated examiners, potentially indicating a tendency towards overestimation as a result of a lack of calibration. In contrast, the change measure (ΔQHI) maintained good reliability confirming that relative change is more robust Against low‐variance conditions. These results collectively suggest that the reliability of plaque indices increases With plaque abundance: The more distinct the deposits, the easier and more reproducible their detection

#### 4.1.2 Plaque Index for Long‐Term Care (PI‐LTC)

The PI‐LTC demonstrated high discriminative capacity, With AUROC values often exceeding 0.90, particularly in total and vestibular scores. Nevertheless, its interexaminer reliability was moderate overall (ICC ≈ 0.50) and varied widely Across tooth groups. Molar regions exhibited the weakest agreement, With ICCs occasionally negative, whereas anterior and premolar regions achieved fair to good reliability (ICC 0.44–0.57). This discrepancy likely results From a combination of factors, including reduced visual access in posterior areas, greater surface complexity, and methodological challenges in standardizing plaque assessment on complex tooth surfaces, particularly when applying less established indices [25, 33]. Additionally, plaque texture and thickness—Rather Than surface staining alone—Seem to play a greater role in PI‐LTC scoring, making it more susceptible to subjective variation. Comparable limitations have been reported by Kharbot et al. [25]. using the buccally assessed GDI‐S on intraoral scans, who likewise observed high diagnostic discrimination but only moderate examiner agreement for plaque assessment. Despite these limitations, aggregated PI‐LTC scores proved highly sensitive and methodologically stable for risk modeling, underlining its potential as a sensitive tool for identifying individuals at elevated plaque risk in domiciliary settings

#### 4.1.3 Modified Gingival Index (MGI)

The MGI achieved moderate to good reliability With better agreement for anterior Than for posterior teeth. Examiner D_2_, who lacked calibration, consistently rated gingival inflammation lower Than the other raters, suggesting that visual assessment of swelling and changes in colour remains inherently examiner‐dependent. Unlike plaque‐based indices, the MGI requires subjective interpretation of color, texture and bleeding [19]—Parameters less easily standardized in digital imaging. Therefore, while the MGI provides complementary information on gingival health, its use in remote or automated digital settings appears more limited Without advanced color calibration or algorithmic image analysis [20]

#### 4.1.4 Comparative Performance of Indices

Direct comparison Between the indices revealed distinct functional strengths. The PI‐LTC showed the highest sensitivity and discrimination (AUROC up to 1.00), making it particularly suitable for identifying high plaque risk. However, its moderate reliability—Especially in molar areas—Limits its use for serial monitoring unless examiner calibration and consistent conditions are ensured and the examiner's focus is set on those areas. Conversely, the QHI achieved slightly lower AUROC values but substantially higher reliability and methodological stability, supporting its application in routine follow‐up or interventional evaluation. The MGI was the most examiner‐dependent index and may require supplementary imaging modalities or AI‐based color segmentation to improve consistency in future digital adaptations

#### 4.1.5 Methodological and Clinical Implications

From a translational standpoint, these findings support the integration of digital intraoral scanning Into domiciliary and community‐based oral health programs as also shown in earlier works [35]. Community‐dwelling older adults represent a particularly relevant target group, as they frequently experience declining oral self‐care capacity while simultaneously facing substantial barriers to accessing professional preventive services [7]. Unlike the general adult population, daily oral hygiene in this group is often supported by informal caregivers or nursing personnel With limited oral health training [6]. As a result, oral hygiene may become a lower priority Within everyday care routines, allowing plaque accumulation and gingival inflammation to progress unnoticed Over extended periods [6, 7]. Several barriers have been reported to limit the provision of oral healthcare for older adults by dentists, including inadequate reimbursement for outreach services, loss of clinical working time, lack of portable equipment and suitable treatment environments, as well as insufficient professional training for the management of frail older patients [15]. These circumstances highlight the need for objective, reproducible, and easily transferable assessment tools that can support both professional and caregiver‐delivered oral healthcare

As access to professional care is often limited [36], digital scans can provide an objective, repeatable record of oral hygiene status. Although intraoral scanners such as the TRIOS 4 system were originally developed for clinical practice settings, their compact design and portability may facilitate future application in domiciliary care environments. In the present study, scans were obtained by one calibrated dental prophylaxis assistant Under standardized conditions, including uniform ambient chair lighting. Because maintaining such standardized conditions may be more challenging in typical home‐care environments, the findings of the present study cannot be directly extrapolated to these settings Additional portable light sources, such as LED headlamps or illumination systems integrated Into magnifying loupes, may help compensate for lower ambient illumination and improve scan acquisition Under outreach conditions [37]. Future studies should therefore investigate the practical performance, scan quality, and diagnostic reliability of intraoral scanning protocols directly Within real‐world domiciliary settings. The data also suggest that standard plaque indices (e.g., QHI) are more robust Under real‐world conditions and could serve as practical endpoints for caregiver‐delivered hygiene programs. For clinical implementation, examiner calibration remains essential, particularly for indices involving subjective interpretation (here PI‐LTC, MGI). However, the fact that even uncalibrated raters achieved acceptable agreement underscores the feasibility of using simplified digital workflows supported by remote expert review. It should be emphasized that scan acquisition in the present study was performed exclusively by a trained dental professional Under standardized conditions. Therefore, the findings cannot be directly extrapolated to non‐dental personnel such as nursing staff, home‐care assistants, or family caregivers, who may represent important operators in future domiciliary care concepts. The feasibility, scan quality, and diagnostic reliability achievable by these user groups require dedicated evaluation Before widespread implementation in community‐based and tele‐dentistry settings can be recommended. The next logical advancement is the integration of artificial intelligence–based image analysis to automatically quantify plaque coverage and gingival changes as it has been done for other diagnoses [38, 39], thereby minimizing human bias and interrater variability. Deep learning approaches trained on annotated intraoral scan datasets for older cohorts could identify plaque distribution and inflammation patterns more consistently Than manual scoring, making continuous digital monitoring of oral hygiene in home care both scalable and objective

## 5 Limitations

Several limitations must be acknowledged. The study was conducted in a single center With a modest sample size (n = 52), which, while sufficient for reliability analyses, limits the generalizability of results. Our study population consisted of community‐dwelling older adults who regularly attended routine dental examinations at a university clinic and were therefore sufficiently fit and motivated to seek dental care Despite their advanced age. This selection most likely explains the low mean number of decayed teeth (0.13), which was lower Than the corresponding estimate reported for 65–74‐year‐olds in the current German Oral Health Study (mean DT 0.4) [40]

All examinations were performed Under controlled conditions, which may not fully replicate the lighting, positioning, and access challenges typical of real domiciliary care environments. The study employed a single intraoral scanner model and software environment; thus, findings may vary With alternative imaging systems. Moreover, the participating examiners, although representing different calibration levels, were all dental professionals, leaving open the question of how non‐dental caregivers might perform Under similar protocols. Furthermore, the risk classification thresholds applied for the PI‐LTC and QHI were adapted From plaque percentage cut‐offs reported in previous literature rather Than being specifically validated for these indices. Although this approach enabled comparative risk modelling, the resulting classifications should be interpreted With caution Until externally validated threshold values become available. The study population consisted of community‐dwelling older adults who were able to attend a university‐based dental clinic. Consequently, the findings may not be fully generalizable to more frail, homebound, or institutionalized populations With greater functional limitations and potentially more challenging oral conditions

## 6 Conclusion

The present findings demonstrate that oral hygiene and gingival health can be assessed on digital intraoral scans With a diagnostic performance comparable to conventional clinical examination. Digital intraoral scanning represents a promising and feasible approach for assessing oral hygiene and gingival health in community‐dwelling older adults. Both the PI‐LTC and QHI demonstrated excellent discriminative capacity, while the QHI additionally offered superior interexaminer reliability. Examiner calibration markedly enhanced measurement precision, particularly for indices involving subjective evaluation such as the MGI and PI‐LTC. Nevertheless, uncalibrated digital assessments also achieved clinically acceptable agreement, supporting their potential utility in outreach or home‐care settings when standardized protocols are followed

Although intraoral scanners currently involve substantial acquisition costs, their long‐term economic value in outreach settings warrants further investigation. In institutional environments such as nursing homes or daycare facilities, a single scanner operated by appropriately trained personnel could enable repeated digital documentation, while scan datasets are remotely evaluated by dental professionals Within a telemedical framework. Such models may improve access to professional oral assessment and reduce logistical and personnel‐related barriers associated With repeated on‐site examinations. However, formal health‐economic analyses are required to determine whether these potential benefits justify the costs of implementation

For future applications, technological refinements such as improved visualization of posterior regions and AI‐assisted plaque quantification may further enhance reproducibility and reduce examiner dependency. Within this framework, the QHI may represent a particularly suitable index for longitudinal digital monitoring, whereas the PI‐LTC may provide additional sensitivity for identifying individuals at increased plaque risk

Taken together, the present findings suggest that digital intraoral scan–based assessment represents a valid and clinically useful adjunct to conventional oral hygiene assessment in older adults. While further studies are required to evaluate real‐world domiciliary implementation, non‐dental operators, and economic aspects, the observed agreement between digital and clinical examination supports the potential role of intraoral scanning w, ithin future tele‐dentistry and interprofessional oral healthcare concepts (Supporting material).

## Author Contributions

Isabel Deeg was the principal author and was primarily responsible for study conception, data interpretation, patient examinations and manuscript preparation. Christoph Matthias Schoppmeier and Karolin Höfer contributed to the evaluation and analysis of imaging scans. Alicia Maria Meyer‐Hofmann conducted patient examinations together with Isabel Deeg. Dirk Bleiel and Anna Greta Barbe provided scientific supervision and methodological guidance throughout the study. All authors critically reviewed the manuscript and approved the final version for publication.

## Ethics Statement

The study received ethical approval from the local ethics review board of the University of Cologne, Cologne, Germany (23‐1060_1, 21.06.2023). All procedures complied with the ethical standards of the institutional research committee, the 1964 Helsinki Declaration, and subsequent amendments or comparable ethical standards. Informed consent was obtained from all participants or their legal guardians.

## Conflicts of Interest

The authors declare no conflicts of interest.

## Supporting information




**Supporting File**: scd70268‐sup‐0001‐Table2.docx.

## Data Availability

The data that support the findings of this study are available from the corresponding author upon reasonable request.
