# Supplementary material for: Reliability and Diagnostic Performance of Digital Versus Clinical Assessment of Oral Hygiene and Gingival Health in Older Community‐dwelling Adults
Source: Spec Care Dentist. 2026 Oct 8;46(5):e70268. doi: 10.1111/scd.70268 (PMC13648007; doi:10.1111/scd.70268)
Supplement: Supplementary file 1 [file SCD-46-0-s001.docx]

Table 2. Inter-examiner reliability and descriptive statistics of PILTC, QHI, and MGI scores

| Variable | Mean | SD | Median | Minimum | Maximum | ICC2 | ICC3 |
| --- | --- | --- | --- | --- | --- | --- | --- |
| PILTC† | | | | | | | |
| PILTC†D0 upper jaw | 0.34 | 0.31 | 0.22 | 0.00 | 1.06 | 0.436 | 0.502 |
| PILTC†D1 upper jaw | 0.38 | 0.30 | 0.28 | 0.05 | 1.08 |  |  |
| PILTC†D2 upper jaw | 0.57 | 0.34 | 0.59 | 0.00 | 1.19 |  |  |
| PILTC†D0 lower Jaw | 0.62 | 0.20 | 0.57 | 0.27 | 1.08 | 0.374 | 0.465 |
| PILTC†D1 lower Jaw | 0.80 | 0.20 | 0.79 | 0.38 | 1.49 |  |  |
| PILTC†D2 lower Jaw | 0.80 | 0.20 | 0.79 | 0.38 | 1.49 |  |  |
| PILTC†D0 oral | 0.57 | 0.18 | 0.54 | 0.27 | 0.95 | 0.402 | 0.533 |
| PILTC†D1 oral | 0.76 | 0.19 | 0.81 | 0.38 | 1.07 |  |  |
| PILTC†D2 oral | 0.76 | 0.19 | 0.81 | 0.38 | 1.07 |  |  |
| PILTC†D0 oral front | 0.55 | 0.42 | 0.58 | 0.00 | 2.00 | 0.516 | 0.544 |
| PILTC†D1 oral front | 0.57 | 0.40 | 0.50 | 0.00 | 1.67 |  |  |
| PILTC†D2 oral front | 0.74 | 0.43 | 0.82 | 0.00 | 2.00 |  |  |
| PILTC†D0 oral molars | -0.24 | 7.06 | 0.70 | -48.50 | 2.00 | -0.022 | -0.022 |
| PILTC†D1 oral molars | 0.81 | 0.63 | 0.80 | -1.20 | 2.00 |  |  |
| PILTC†D2 oral molars | 0.83 | 0.43 | 1.00 | 0.00 | 2.00 |  |  |
| PILTC†D0 oral premolars | 0.58 | 0.48 | 0.60 | 0.00 | 1.75 | 0.500 | 0.524 |
| PILTC†D1 oral premolars | 0.59 | 0.46 | 0.60 | 0.00 | 1.50 |  |  |
| PILTC†D2 oral premolars | 0.77 | 0.43 | 0.85 | 0.00 | 1.75 |  |  |
| PILTC†D0 buccal | 0.42 | 0.35 | 0.29 | 0.00 | 1.32 | 0.533 | 0.592 |
| PILTC†D1 buccal | 0.63 | 0.34 | 0.59 | 0.00 | 1.33 |  |  |
| PILTC†D2 buccal | 0.63 | 0.34 | 0.59 | 0.00 | 1.33 |  |  |
| PILTC†D0 buccal front | 0.39 | 0.41 | 0.28 | 0.00 | 1.50 | 0.571 | 0.612 |
| PILTC†D1 buccal front | 0.46 | 0.42 | 0.33 | 0.00 | 1.88 |  |  |
| PILTC†D2 buccal front | 0.61 | 0.37 | 0.67 | 0.00 | 1.25 |  |  |
| PILTC†D0 buccal molarss | 0.62 | 0.48 | 0.50 | 0.00 | 2.00 | 0.225 | 0.226 |
| PILTC†D1 buccal molars | 0.66 | 0.49 | 0.60 | 0.00 | 2.00 |  |  |
| PILTC†D2 buccal molars | 0.73 | 0.46 | 0.75 | 0.00 | 2.00 |  |  |
| PILTC†D0 buccal premolars | 0.28 | 0.35 | 0.14 | 0.00 | 1.50 | 0.443 | 0.502 |
| PILTC†D1 buccal premolars | 0.41 | 0.44 | 0.25 | 0.00 | 1.75 |  |  |
| PILTC†D2 buccal premolars | 0.58 | 0.42 | 0.50 | 0.00 | 1.75 |  |  |
| PILTC†D0 total | 0.56 | 0.44 | 0.46 | 0.00 | 2.30 | 0.533 | 0.560 |
| PILTC†D1 total | 0.58 | 0.40 | 0.44 | 0.06 | 1.90 |  |  |
| PILTC†D2 total | 0.75 | 0.45 | 0.70 | 0.00 | 2.58 |  |  |
| QHI^‡^ | | | | | | | |
| QHI^‡^pre D0 upper jaw | 1.36 | 0.66 | 1.38 | 0.27 | 3.05 | 0.721 | 0.722 |
| QHI^‡^pre D1 upper jaw | 1.43 | 0.68 | 1.50 | 0.33 | 2.92 |  |  |
| QHI^‡^pre D2 upper jaw | 1.33 | 0.61 | 1.29 | 0.09 | 3.00 |  |  |
| QHI^‡^pre D0 lower jaw | 1.83 | 0.80 | 1.71 | 0.13 | 3.50 | 0.651 | 0.654 |
| QHI^‡^pre D1 lower jaw | 1.82 | 0.79 | 1.74 | 0.05 | 3.43 |  |  |
| QHI^‡^pre D2 lower jaw | 1.68 | 0.65 | 1.61 | 0.00 | 3.58 |  |  |
| QHI^‡^pre D0 oral | 1.68 | 0.76 | 1.62 | 0.21 | 3.21 | 0.639 | 0.641 |
| QHI^‡^pre D1 oral | 1.69 | 0.78 | 1.64 | 0.31 | 3.22 |  |  |
| QHI^‡^pre D2 oral | 1.56 | 0.66 | 1.50 | 0.00 | 3.03 |  |  |
| QHI^‡^pre D0 oral front | 1.70 | 0.80 | 1.77 | 0.00 | 3.60 | 0.664 | 0.666 |
| QHI^‡^pre D1 oral front | 1.64 | 0.80 | 1.58 | 0.40 | 3.75 |  |  |
| QHI^‡^pre D2 oral front | 1.56 | 0.73 | 1.58 | 0.00 | 3.44 |  |  |
| QHI^‡^pre D0 oral molars | 1.67 | 0.76 | 1.62 | 0.28 | 3.36 | 0.625 | 0.625 |
| QHI^‡^pre D1 oral molars | 1.78 | 0.99 | 1.70 | 0.00 | 4.00 |  |  |
| QHI^‡^pre D2 oral molars | 1.70 | 0.86 | 1.82 | 0.00 | 3.50 |  |  |
| QHI^‡^pre D0 oral premolars | 1.54 | 0.88 | 1.40 | 0.00 | 3.25 | 0.546 | 0.547 |
| QHI^‡^pre D1 oral premolars | 1.59 | 0.83 | 1.43 | 0.20 | 3.50 |  |  |
| QHI^‡^pre D2 oral premolars | 1.41 | 0.73 | 1.38 | 0.00 | 3.00 |  |  |
| QHI^‡^pre D0 buccal | 1.57 | 0.72 | 1.51 | 0.20 | 3.43 | 0.720 | 0.727 |
| QHI^‡^pre D1 buccal | 1.63 | 0.73 | 1.60 | 0.46 | 3.55 |  |  |
| QHI^‡^pre D2 buccal | 1.46 | 0.65 | 1.43 | 0.09 | 4.17 |  |  |
| QHI^‡^pre D0 buccal front | 1.56 | 0.80 | 1.52 | 0.08 | 3.25 | 0.723 | 0.726 |
| QHI^‡^pre D1 buccal front | 1.54 | 0.78 | 1.44 | 0.17 | 3.50 |  |  |
| QHI^‡^pre D2 buccal front | 1.43 | 0.65 | 1.33 | 0.00 | 3.60 |  |  |
| QHI^‡^pre D0 buccal molars | 1.63 | 0.87 | 1.57 | 0.00 | 3.40 | 0.667 | 0.668 |
| QHI^‡^pre D1 buccal molars | 1.85 | 0.97 | 1.83 | 0.00 | 4.00 |  |  |
| QHI^‡^pre D2 buccal molars | 1.63 | 0.86 | 1.58 | 0.00 | 3.57 |  |  |
| QHI^‡^pre D0 buccal premolars | 1.44 | 0.73 | 1.43 | 0.12 | 3.33 | 0.613 | 0.636 |
| QHI^‡^pre D1 buccal premolars | 1.59 | 0.83 | 1.62 | 0.00 | 3.50 |  |  |
| QHI^‡^pre D2 buccal premolars | 1.25 | 0.72 | 1.21 | 0.00 | 4.00 |  |  |
| QHI^‡^pre D0 total | 1.63 | 0.69 | 1.54 | 0.12 | 3.12 | 0.741 | 0.750 |
| QHI^‡^pre D1 total | 1.70 | 0.68 | 1.74 | 0.52 | 3.33 |  |  |
| QHI^‡^pre D2 total | 1.53 | 0.53 | 1.50 | 0.26 | 3.16 |  |  |
| QHI^‡^post D0 oral front | 0.01 | 0.03 | 0.00 | 0.00 | 0.17 | 0.008 | 0.009 |
| QHI^‡^post D1 oral front | 0.02 | 0.05 | 0.00 | 0.00 | 0.25 |  |  |
| QHI^‡^post D2 oral front | 0.13 | 0.26 | 0.00 | 0.00 | 1.60 |  |  |
| QHI^‡^post D0 oral molars | 0.01 | 0.05 | 0.00 | 0.00 | 0.33 | 0.095 | 0.115 |
| QHI^‡^post D1 oral molars | 0.07 | 0.17 | 0.00 | 0.00 | 1.00 |  |  |
| QHI^‡^post D2 oral molars | 0.37 | 0.68 | 0.11 | 0.00 | 3.00 |  |  |
| QHI^‡^post D0 oral premolars | 0.01 | 0.06 | 0.00 | 0.00 | 0.33 | 0.050 | 0.059 |
| QHI^‡^post D1 oral premolars | 0.02 | 0.05 | 0.00 | 0.00 | 0.25 |  |  |
| QHI^‡^post D2 oral premolars | 0.18 | 0.38 | 0.00 | 0.00 | 2.00 |  |  |
| QHI^‡^post D0 buccal front | 0.02 | 0.14 | 0.00 | 0.00 | 1.00 | -0.041 | -0.045 |
| QHI^‡^post D1 buccal front | 0.01 | 0.05 | 0.00 | 0.00 | 0.25 |  |  |
| QHI^‡^post D2 buccal front | 0.11 | 0.23 | 0.00 | 0.00 | 1.12 |  |  |
| QHI^‡^post D0 buccal molars | 0.01 | 0.03 | 0.00 | 0.00 | 0.17 | 0.031 | 0.038 |
| QHI^‡^post D1 buccal molars | 0.14 | 0.26 | 0.00 | 0.00 | 1.00 |  |  |
| QHI^‡^post D2 buccal molars | 0.38 | 0.67 | 0.17 | 0.00 | 3.00 |  |  |
| QHI^‡^post D0 buccal premolars | 0.00 | 0.03 | 0.00 | 0.00 | 0.20 | 0.115 | 0.129 |
| QHI^‡^post D1 buccal premolars | 0.03 | 0.08 | 0.00 | 0.00 | 0.33 |  |  |
| QHI^‡^post D2 buccal premolars | 0.12 | 0.28 | 0.00 | 0.00 | 1.50 |  |  |
| QHI^‡^post D0 total | 0.01 | 0.02 | 0.00 | 0.00 | 0.11 | 0.033 | 0.047 |
| QHI^‡^post D1 total | 0.03 | 0.04 | 0.00 | 0.00 | 0.15 |  |  |
| QHI^‡^post D2 total | 0.16 | 0.22 | 0.10 | 0.00 | 1.13 |  |  |
| ΔQHI^§^D0 total | -1.60 | 0.65 | -1.57 | -3.00 | -0.20 | 0.640 | 0.677 |
| ΔQHI^§^D1 total | -1.62 | 0.68 | -1.62 | -2.91 | -0.30 |  |  |
| ΔQHI^§^D2 total | -1.34 | 0.52 | -1.27 | -2.33 | -0.05 |  |  |
| MGI^¶^ | | | | | | | |
| MGI^¶^D0 upper jaw | 0.97 | 0.72 | 0.76 | 0.00 | 3.75 | 0.547 | 0.657 |
| MGI^¶^D1 upper jaw | 1.01 | 0.72 | 0.82 | 0.08 | 3.75 |  |  |
| MGI^¶^D2 upper jaw | 0.49 | 0.43 | 0.38 | 0.00 | 2.00 |  |  |
| MGI^¶^D0 lower jaw | 1.05 | 0.69 | 0.90 | 0.04 | 2.81 | 0.511 | 0.639 |
| MGI^¶^D1 lower jaw | 1.07 | 0.66 | 0.86 | 0.11 | 2.92 |  |  |
| MGI^¶^D2 lower jaw | 0.55 | 0.30 | 0.50 | 0.03 | 1.50 |  |  |
| MGI^¶^D0 oral | 1.02 | 0.71 | 0.87 | 0.00 | 3.58 | 0.508 | 0.650 |
| MGI^¶^D1 oral | 1.03 | 0.65 | 0.90 | 0.05 | 3.46 |  |  |
| MGI^¶^D2 oral | 0.47 | 0.41 | 0.34 | 0.00 | 2.00 |  |  |
| MGI^¶^D0 oral front | 0.87 | 0.68 | 0.73 | 0.00 | 2.83 | 0.563 | 0.657 |
| MGI^¶^D1 oral front | 0.91 | 0.65 | 0.76 | 0.00 | 2.83 |  |  |
| MGI^¶^D2 oral front | 0.46 | 0.44 | 0.33 | 0.00 | 2.00 |  |  |
| MGI^¶^D0 oral molars | 1.23 | 0.75 | 1.00 | 0.00 | 3.00 | 0.438 | 0.627 |
| MGI^¶^D1 oral molars | 1.23 | 0.72 | 1.12 | 0.00 | 3.00 |  |  |
| MGI^¶^D2 oral molars | 0.50 | 0.48 | 0.40 | 0.00 | 2.00 |  |  |
| MGI^¶^D0 oral premolars | 1.05 | 0.71 | 1.00 | 0.00 | 3.00 | 0.438 | 0.577 |
| MGI^¶^D1 oral premolars | 1.01 | 0.65 | 0.86 | 0.00 | 3.00 |  |  |
| MGI^¶^D2 oral premolars | 0.43 | 0.42 | 0.35 | 0.00 | 1.75 |  |  |
| MGI^¶^D0 buccal | 1.03 | 0.71 | 0.91 | 0.00 | 2.92 | 0.519 | 0.600 |
| MGI^¶^D1 buccal | 1.06 | 0.71 | 0.82 | 0.16 | 3.21 |  |  |
| MGI^¶^D2 buccal | 0.61 | 0.38 | 0.54 | 0.04 | 2.00 |  |  |
| MGI^¶^D0 buccal front | 0.98 | 0.78 | 0.75 | 0.00 | 3.00 | 0.548 | 0.602 |
| MGI^¶^D1 buccal front | 0.99 | 0.77 | 0.67 | 0.00 | 3.00 |  |  |
| MGI^¶^D2 buccal front | 0.60 | 0.44 | 0.59 | 0.00 | 2.00 |  |  |
| MGI^¶^D0 buccal molars | 0.95 | 0.65 | 1.00 | 0.00 | 2.50 | 0.462 | 0.538 |
| MGI^¶^D1 buccal molars | 1.06 | 0.65 | 1.00 | 0.00 | 2.50 |  |  |
| MGI^¶^D2 buccal molars | 0.64 | 0.56 | 0.50 | 0.00 | 3.00 |  |  |
| MGI^¶^D0 buccal premolars | 1.01 | 0.74 | 0.88 | 0.00 | 2.75 | 0.478 | 0.563 |
| MGI^¶^D1 buccal premolars | 1.02 | 0.70 | 0.80 | 0.00 | 2.88 |  |  |
| MGI^¶^D2 buccal premolars | 0.55 | 0.39 | 0.50 | 0.00 | 1.75 |  |  |
| MGI^¶^D0 total | 1.03 | 0.72 | 0.91 | 0.06 | 3.15 | 0.508 | 0.594 |
| MGI^¶^D1 total | 1.03 | 0.63 | 0.88 | 0.17 | 3.25 |  |  |
| MGI^¶^D2 total | 0.58 | 0.50 | 0.46 | 0.03 | 2.69 |  |  |

Descriptive statistics and inter-examiner reliability of oral health indices assessed independently by three examiners (D0, D1, D2); D0 = clinical examiner (reference); D1 = calibrated digital examiner; D2 = non-calibrated digital examiner

The Plaque Index for Long-Term Care (PILTC), Quigley-Hein Plaque Index (QHI) before and after professional brushing procedures, change in QHI (ΔQHI), and the Modified Gingival Index (MGI) were recorded for different jaw regions and tooth groups. Data are presented as mean, standard deviation (SD), median, minimum, maximum, and intraclass correlation coefficients (ICC2 and ICC3).

ICC2 = two-way random-effects model, absolute agreement.
ICC3 = two-way mixed-effects model, consistency.

SD = standard deviation.

According to Cicchetti^1^, intraclass correlation coefficients were interpreted as follows and are visually coded in the table:
ICC < 0.40 = poor reliability (red),
ICC 0.40–0.59 = fair reliability (yellow),
ICC 0.60–0.74 = good reliability (green),
ICC ≥ 0.75 = excellent reliability (dark green).

^†^ PILTC= Plaque Index for Long Term Care
^‡^ QHI= Quigley-Hein Index.
^§^ ΔQHI= Change in QHI from pre- to post-professional brushing
^¶^ MGI = Modified Gingival Index

1.	Cicchetti DV. Guidelines, criteria, and rules of thumb for evaluating normed and standardized assessment instruments in psychology. *Psychological Assessment* 1994;6:284–290.
